# Changes in disc status in the reducing and nonreducing anterior disc displacement of temporomandibular joint: a longitudinal retrospective study

**DOI:** 10.1038/srep34253

**Published:** 2016-09-27

**Authors:** Ying Kai Hu, Chi Yang, Qian Yang Xie

**Affiliations:** 1 Department of Oral Surgery, Shanghai Ninth People’s Hospital, Shanghai Jiao Tong University, School of Medicine, Shanghai, Shanghai Key Laboratory of Stomatology, People’s Republic of China

## Abstract

Treatment procedures for anterior disc displacement (ADD) of temporomandibular joint (TMJ) are far from reaching a consensus. The aim of the study was to evaluate disc status changes of anterior disc displacement with reduction (ADDWR) and without reduction (ADDWoR) comparatively, to get a better understanding of the disease progress without intervention. This longitudinal retrospective study included 217 joints in 165 patients, which were divided into ADDWR group and ADDWoR group based on magnetic resonance imaging (MRI) examination. The joints were assessed quantitatively for disc length and displacement distance at initial and follow-up visits. Disc morphology, which was classified in 5 types, was also evaluated. Paired t-test and Wilcoxon signed rank test were used to assess intra-group differences and independent t-test for inter-group differences. Moreover, analysis of covariance was applied to analyze influential factors for changes in disc length and displacement distance. According to our results, discs tended to become shorter, move further forward and distort more seriously in ADDWoR group than in ADDWR group after follow-up. Moreover, discs were prone to become shorter and more anteriorly displaced in teenagers, type I and III morphologies, advanced Wilkes stages, or those with joint effusion. Follow-up period seemed to be not critical.

Anterior disc displacement (ADD) is often observed in patients seeking treatment for the symptoms of temporomandibular joint (TMJ), including anterior disc displacement with reduction (ADDWR) and anterior disc displacement without reduction (ADDWoR). It occurs in people of all ages, with a high prevalence in women aged 20 to 40 years, resulting in clicking, joint pain, limited range of mouth opening, masticatory difficulty, mandible dysfunction, and so on^1^. Many options have been suggested for the management of ADD of the TMJ, such as medications, occlusal appliances, physical therapy, arthrocentesis, arthroscopic lysis or lavage and disc repositioning by arthroscopy or open surgery^2^,^3^. However, the treatment procedures are far from reaching a consensus. Some researchers consider that clinical symptoms and dysfunction of TMJ internal derangement (ID) tend to subside with time, conservative therapy, even abandonment of any therapy have been proposed^4^,^5^, and arthrocentesis and arthroscopic lysis and lavage also seem to relieve pain and dysfunction^6^, yet repositioning the disc is neither a goal nor a result of these procedures. Whiles others believe that disc position is important, despite clinical improvements, progressive degenerative joint disease still can be detected, even disturbing facial growth and developing mandibular asymmetry, and it is significantly related to ADD, especially to ADDWoR^7^,^8^,^9^,^10^,^11^,^12^, thus an early disc repositioning is necessary. Therefore, an understanding of the disease progress without intervention is prerequisite for evaluating the true effect of a particular treatment.

Magnetic resonance imaging (MRI), as a non-invasive, radiation-free imaging technique with superior soft-tissue resolution, has been described as the gold standard for the examination of TMJ^13^,^14^,^15^. It can assess the integrity of hard and soft tissue inside the TMJ, to grade the severity of the ID by showing the morphology of the disc^16^.

Several studies have examined the natural course of disc position and configuration in ADDWoR^2^,^4^,^17^,^18^,^19^, showing persistent existence of disc displacement, continued disc deformity and probably accelerated bone change. Besides, our previous study showed the disc would likely become more anteriorly displaced and shortened, and ADDWR could turn into ADDWoR^17^. The purposes of this study were to quantitatively measure disc length, displacement distance, disc morphology between ADDWR and ADDWoR, as well as analyzing the influential factors of changes in disc status, to achieve more insights into the natural course of ADD of the TMJ. The authors hypothesized that changes in disc length and displacement distance would be greater in ADDWoR than ADDWR, so was the deformity extension of disc.

## Materials and Methods

### Study design

This was a longitudinal retrospective study, with approval of the institutional review board of Shanghai Ninth People’s Hospital, Shanghai Jiao Tong University School of Medicine. The study followed the tenets of the Declaration of Helsinki for research involving human subjects, and all participants signed an informed consent agreement.

The patients included in this retrospective study were collected from a consecutive series of patients who had been referred to the TMJ division of the Department of Oral Surgery in Shanghai Ninth People’s Hospital affiliated to Shanghai Jiao Tong University School of Medicine. The inclusion and exclusion criteria were presented in Table 1.

### Study variables

The predictor variable was the ADDWR versus the ADDWoR. The outcome variables were changes in disc length, displacement distance over time. Other variables consisted of age, gender, follow-up period, stage of ID, disc morphology, and joint effusion.

In this study, disc length (L) and displacement distance (D) were measured directly. L_1_ and D_1_ were defined as disc length and displacement distance at initial visit, while L_2_ and D_2_ were those at follow-up visit. Changes in disc length (∆L) and displacement distance (∆D) were computed (∆L = L2 − L1; ∆D = D2 − D1), indicating the changes during the interval.

### MRI of TMJ

MRI scans were obtained using a 1.5-T imager (Signa, General Electric, Milwaukee, WI) with bilateral 3-inch TMJ surface coil receivers. Details about the TMJ MRI scans obtained in the clinic were as following. 1) The transection plane was scanned to find the long axis of the condyle. The sagittal plane was then determined to be perpendicular to this long axis, because the coronal plane paralleled the long axis. 2) The T1-weighted spin echo sequence in the sagittal plane in a closed-mouth position was scanned. 3) The T1-weighted spin echo sequence in the coronal plane was scanned at closed-mouth position. 4) The T2-weighted spin echo sequence in the sagittal plane was scanned at open-mouth position. The parameters for the T1-weighted spin echo sequence on the sagittal and coronal images were a repetition time of 1,800 millseconds, an echo time of 20 millseconds, 2 excitations, and a field of view of 12 cm. A slice thickness of 1 mm with a skip of 0.3 mm and a matrix of 512 × 256 pixels was used. The parameters for the T2-weighted spin echo sequence on the sagittal images were the same as those for the T1-weighted spin echo sequence, except for a repetition time of 3,800 millseconds and an echo time of 80 millseconds^3^.

### Evaluation of MRI

Quantitative measurements were performed by two oral and maxillofacial surgeons, and remeasured at a 2-week interval. Intra- and inter-examiner reliabilities were estimated. Furthermore, categorical variables were assessed by the same two surgeons. When there was a disagreement, consensus was reached by discussion.

The sagittal slice with the largest cross-section of the condyle (usually the central slice) was chosen for tracing, on which some points and reference lines were drawn with the assistance of Adobe Photoshop CS5 (Adobe Systems, San Jose, CA). Next, linear measurements for disc length and displacement distance were achieved using MB-Ruler measuring software (accurate to 0.01 mm) (Markus Bader, Berlin, Germany).

#### Stage of ID

Based on Wilkes and Bronstein’s classification criteria^20^, the TMJ ID was classified into 5 stages: stage I: simple disk displacement without changes in disc morphology or osteoarthrosis (OA); stage II: reducible disc displacement with mild-moderate deformity of the disc and/or OA; stage III: permanent disc displacement with mild to moderate disc deformity and/or OA; stage IV: sever permanent disc displacement and deformity with OA; stage V: sever permanent disc displacement, associated with disc perforation and obvious disc deformation, as well as OA.

#### Disc length and displacement distance

The long axis of the condyle was determined by a two-step method described by Nebbe *et al*. and Xie *et al*.^21^,^22^. First, the largest circle (*O*_*1*_) internally tangent to the outline of the anterior, posterior, and superior surfaces of the condylar head, allowing separation of the condylar head from the neck region. Second, an internally tangent circle (*O*_*2*_) was drawn at the most curved area between the condylar head and neck. A line joining those two circle centers defined the long axis of the condylar head (*y*).

**Figure 1:**
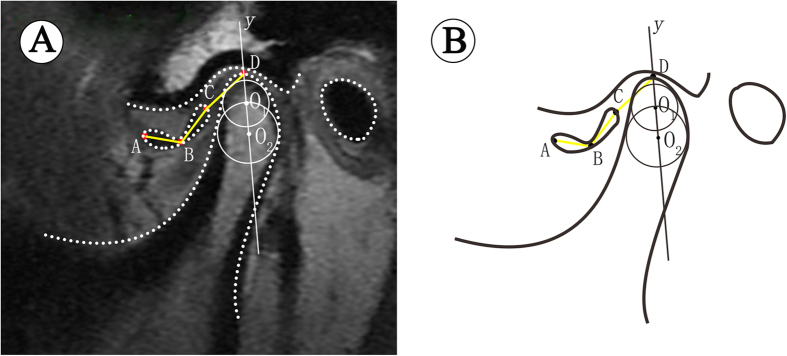
Measurement of disc length and displacement distance on MRI. (**A**) In TMJ MRI image, (**B**) Schematic diagram.

Points A and C were the most anterior and most posterior points of the disc corresponding to the long axis of the condylar head. Point B was the midpoint of the intermediate zone of the disc. Point D was the intersection point where *y* crossed the condylar outline. Linear measurement was performed for AB and BC, and the summation of these 2 distances was defined as the disc length (Fig. 1). If the disc was obviously deformed and the intermediate zone could not be distinguished, the distance of AC was measured directly. Moreover, the distance of C and D was used to determine the displacement distance relative to the condyle^17^,^22^.

#### Disc morphology

**Figure 2:**
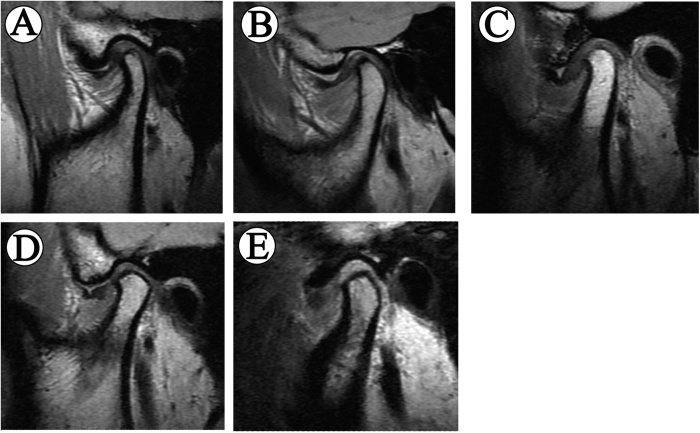
Different types of disc morphology. (**A**) Type I: biconcave shape, (**B**) Type II: biconcave shape with pseudo-disc changes, (**C**) Type III: V-shaped folded configuration without distinct reduce in disc length, (**D**) Type IV: folded and shortened disc with inadequate length to cover the condylar head, (**E**) Type V: rounded configuration.

Disc morphology was evaluated in the closed-mouth position and classified into 5 types (Fig. 2): type I: biconcave configuration with no deformity; type II: biconcave configuration with thick posterior band or mildly folded; type III: moderate folded, U-shaped or V-shaped disc with sufficient length to cover the condylar head; type IV: folded and shortened disc with inadequate length to cover the condylar head; type V: severely folded, biconvex or rounded configuration.

#### Joint effusion

Joint effusion was recorded as present or absent, which was identified when high signal intensity in joint space was seen on T2-weighted imaging. When more than 1 line of high signal was evident in at least 2 consecutive sections, it was considered positive for joint effusion^23^.

### Statistical analysis

The data were analyzed by standard statistical software packages (SPSS, version 17.0, Chicago). A *P* value of less than 0.05 was accepted as statistically significant.

Disc length and displacement distance were compared before and after follow-up in ADDWR and ADDWoR groups using paired t-test, while Wilcoxon signed rank test was used to assess the differences in Wilkes stage and disc morphology. Differences in ∆L and ∆D between the two groups were analyzed using independent t-test.

A preliminary analysis was accomplished using univariate analysis to study the influence of contributing factors for ∆L and ∆D instead of directly applying analysis of covariance. Those variables achieving a *P* value of less than 0.10 were enrolled in the multivariate analysis. The candidate contributing factors were gender, age, follow-up period, Wilkes stage, disc morphology, and joint effusion. Age and follow-up period were categorized: 1) age: 1 = < 15 years, 2 = 15 ≤ age < 22 years, and 3 = ≥ 22 years; 2) follow-up period (F): 1 = 3 ≤ F ≤ 6 months, 2 = 6 < F ≤ 12 months, and 3 = >12 months.

## Results

### Description of the patients

A total of 225 joints were enrolled in the present study, but 8 were excluded (6 for ADDWR turning into ADDWoR and 2 for poor MRI images), so the final total was 217 joints in 165 patients (130 females and 35 males), whose ages ranged from 11 to 64 years at the initial visit (mean, 20.76 years). On initial MRI examination, ADDWR was verified in 86 joints and ADDWoR in 131 joints. The mean follow-up interval of the ADDWR group was 9.38 months (range 3.17–25.47 months) and the ADDWoR group was 8.03 months (range 3.40–47.43 months) (Table 2).

### MRI evaluations

The average disc length significantly decreased from 8.29 mm to 7.21 mm (*P* < 0.001), and the average displacement distance significantly increased from 5.29 mm to 6.40 mm (*P* < 0.001). The disc was shorter and more anteriorly displaced in the ADDWoR group than the ADDWR group both at initial and follow-up visits (*P* < 0.001). Changes in disc length and displacement distance were significantly greater in the ADDWoR group than those of the ADDWR group (*P* < 0.05) (Table 3). Intraclass correlation coefficients (ICCs) for inter-observer agreement was ranged between 0.85 and 0.89, and intra-observer agreement was ranged between 0.96 and 0.99, demonstrating excellent reliabilities.

As revealed by Wilcoxon signed rank test, significant differences in disc morphology and Wilkes stage were found between the initial and follow-up visits in both groups. The disc continued to be more anteriorly displaced and tended to deteriorate with time, the degenerative changes in condylar bones could also be observed. The types of disc morphology differed significantly (*P* < 0.01) intra- and inter- groups at initial visit and follow-up visit. Type I was the primary type of the ADDWR group at initial and follow-up visits, while the predominant type in the ADDWoR group progressed from type II (49.6%) to type IV (40.5%). As far as the Wilkes stage was concerned, 26 out of 86 joints turned from stage I to stage II in the ADDWR group, whereas stage IV (56.5%) became the major component at follow-up visit instead of stage III (75.6%) at initial visit in the ADDWoR group (Table 4).

There was no significant difference in joint effusion between the two groups both at initial and follow-up visits (*P* = 0.118, 0.263, respectively) (Table 5).

### Analysis of risk factors

Among the between-subjects factors examined, gender, age, disc morphology, and joint effusion exhibited a statistically significant association (*P* < 0.01) with ∆L and ∆D. On the contrary, follow-up period did not seem to play an important role in changes of disc length and displacement distance (*P* > 0.1). Although Wilkes stage had influence on change of disc length (*P* < 0.001), its effect on change of displacement distance was questionable (*P* = 0.046) (Table 6).

**Figure 3:**
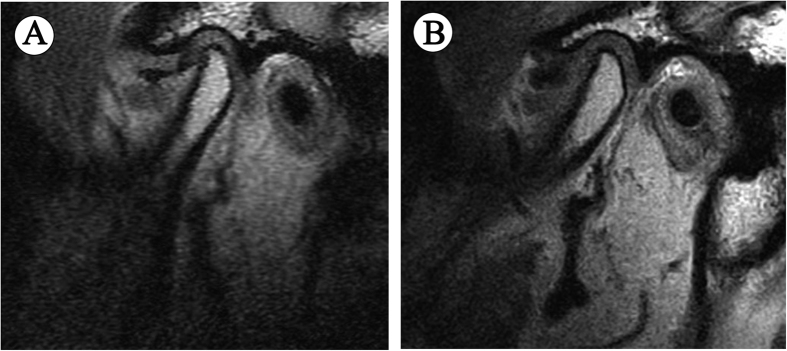
MRI scans of a 55-year-old male ADDWoR patient with the interval of 11 months, showing significant deterioration of disc status. (**A**) First visit, (**B**) Follow-up visit.

**Figure 4:**
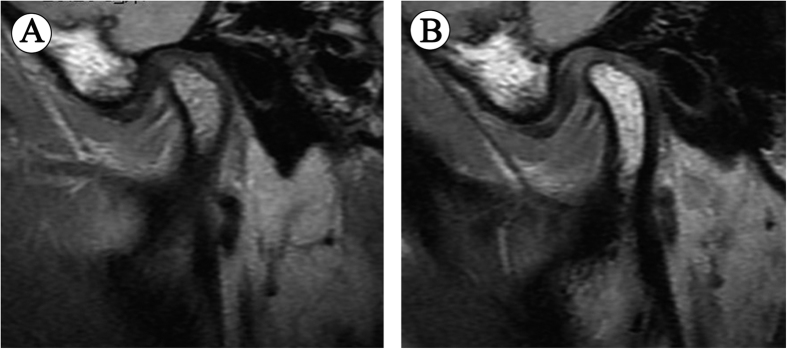
MRI scans of a 16-year-old female ADDWR patient with the interval of 21 months, showing almost unchanged disc status. (**A**) First visit, (**B**) Follow-up visit.

When the analysis of covariance was applied for ∆L, age, disc morphology, Wilkes stage and joint effusion were correlated (Table 7), whereas gender was categorized as nonsignificant. The younger the patient was, the more the disc shortened (*P* ≤ 0.001). In terms of disc morphology, unlike the results of univariate analysis, compared with type I, the danger of disc shortening decreased significantly in type II, IV and V (*P* = 0.025, *P* < 0.001, and *P* = 0.021, respectively), while no significant difference was found between type I and III (*P* = 0.135), demonstrating U-shaped or V-shaped folded configuration accelerated disc shortening. Besides, the disc shortened more in ADDWoR (stage III–V) (Fig. 3) than in ADDWR (stage I and II) (Fig. 4). Furthermore, joint effusion seemed to raise the risk of disc shortening significantly, too (*P* = 0.002).

As for displacement distance changes, gender, age, disc morphology, Wilkes stage and joint effusion were related (Table 8). Disc tended to be more anteriorly displaced in male than female (*P* = 0.031), and in adolescents than adults (*P* < 0.001). Type IV and V morphologies manifested less change in displacement distance, but significant difference between type V and type I was questionable (*P* = 0.051). Further statistically significant effects emerged, when Wilkes stage and joint effusion were related to ∆D (*P* < 0.05, and *P* = 0.029, respectively). Disc in ADDWoR was predisposed to move further forward than in ADDWR. In addition, presence of joint effusion was a risk factor for ∆D.

## Discussion

On basis of MRI, the present study evaluated the differences in changes in disc status, including disc length, position and morphology, between the ADDWR and ADDWoR groups through quantitative and qualitative measurements, as well as analyzing the factors influencing the changes in disc length and position relative to the condyle. To our knowledge, it is the first time that quantitative measurements were performed to compare the differences in disc status between ADDWR and ADDWoR. The results confirmed the authors’ hypothesis, which compared with ADDWR, disc status became worse in ADDWoR, and it might be associated with age, gender, disc morphology, Wilkes stage and joint effusion.

MRI is a non-invasive and non-radioactive imaging technique with superior soft-tissue resolution, and has proven to be the most accurate exam for evaluating the position of the TMJ disc, being described as the “gold standard” for this purpose^24^,^25^. It was reported that the MRI diagnostic accuracies of disc displacement and deformity to be 80% to 90%^26^. Hence, measurements on MRI images would be of high precision.

An incorrectly positioned disc is not indicative of symptoms of TMJ disorders, and the severity of alterations on the imaging exams may not related to the severity of clinical findings, such as pain or mouth opening^27^. Some studies have shown that the clinical symptoms of ADD tend to be alleviated during the natural course^4^,^5^,^19^. However, the disc would continue to be anteriorly displaced and deformation is commonly observed, including folding, enlargement of the posterior band, even thickness and convexity^2^. At a late stage of TMJ disorders, disc perforation may occur more frequently, and what’s more, bone degeneration, facial skeletal changes and occlusal deformities as the result of condylar bone loss and diminished mandibular growth secondary to a displaced disc have been reported^10^,^28^,^29^. One should take those into consideration when anterior disc displacement of the TMJ is treated.

In a study investigated by Sato *et al*.^2^, the disc became displaced further forward, with greater disc deformity in the natural course of nonreducing disc displacement in the TMJ, during a mean follow-up of 27.1 months. Our results were in accordance with previous study. In our study, the mean follow-up interval of the ADDWR group was 9.38 months and the ADDWoR group was 8.03 months. Although disc length and position manifested significant changes in both groups during follow-up period, they got worse in ADDWoR than in ADDWR. The disc length in the ADDWoR group reduced by 1.36 mm, more than that in the ADDWR group which was 0.66 mm. Moreover, the displacement distance of ADDWoR patients was about 0.5 mm longer than that of ADDWR patients. As regards disc morphology, when a biconcave appearance (type I and type II) of the disc was considered normal, most of ADDWR patients had normal configuration at initial and follow-up visits (82.6%, 77.9%, respectively), while proportion of disc deformation in the ADDWoR group increased from 38.2% to 58%, in which folded and shortened disc was common. Patients of ADDWoR group experienced greater deterioration of disc status, with shorter length, longer distance to the condyle and severer distortion, even with a shorter follow-up period than those of ADDWR group. This may result from repeating impingement of the condyle during opening and closing movement in nonreducing conditions, while the disc returning to a normal position in ADDWR during opening movement decreases impact force.

Cortical bone begins to form around the periphery of the condyle during 12 to 14 years old, and by the age of 21–22, full development of the mandibular condyle is accomplished^30^. Disc morphology was classified into 4 types (biconcave, enlargement of the posterior band, even thickness, and biconvex) or 5 types (biconcave, thick posterior band, lengthened, biconvex, folded, and rounded) in previous reports^16^,^31^. Based on pertinent literatures, all the data was divided into 3 subgroups by age, 3 by follow-up period, 5 by disc morphology, 4 by Wilkes stage (stage IV and V were merged), and 2 by joint effusion. This was deemed sufficient to apply analysis of covariance, not only because the incapability of univariate analysis for simultaneous evaluation of all factors, but also to eliminate the interference of confounding factors, which could achieve a more accurate result. The multivariate analysis indicated that age, ADDWoR and joint effusion were risk factors for disc length and displacement distance. In addition, greater changes in disc length were found in type I and III morphologies, yet type IV and type V morphology had less changes in displacement distance. The female: male ration (3.7:1) in this study is well in line with the gender distribution in other studies^32^,^33^. However, gender seemed to influence ∆D, but not ∆L.

An altered disc morphology has been recognized as an important feature of ID, which is frequently found in ADDWoR, and the normal biconcave configuration would like to change as a result of the displacement^31^. It is naturally to think that the closer the disc is to the condyle, the higher compression it would get during the condyle movement, leading to more shrinkage of its length. The distance to the condyle of type III morphology was closer than that of type IV and type V, together with its U-shaped or V-shaped folded configuration, it would get higher compression during the condyle movement. Notwithstanding type I and type II morphologies dominated in ADDWR, remodeling of the posterior disc attachment was absent in type I, resulting in lower capability to bear the load. Consequently, more distinct decrease in disc length was found in type I and III morphologies. Type IV and V morphologies usually accompany with a relatively long duration, so the posterior disc attachment is close to full stretch and the flexibility tends to reduce, which may account for less changes in displacement distance, despite nonsignificant difference between type V and type I.

It has been reported that TMJ effusion indicates synovitis, representing an inflammatory response to a dysfunctional disc-condyle relationship, which was found with a higher probability in ADDWoR compared to ADDWR, and connected with disc deformation^32^,^33^,^34^,^35^. Frequency of joint effusion tends to reduce after period of time, which may be associated with pain relief^35^,^36^. As shown in Table 5, prevalence of effusion decreased during follow-up period, and there was no significant difference between the two groups. On one hand, effusion would reduce spontaneously with time, on the other hand, the reduction might be due to the anti-inflammatory drugs that patients took during follow-up period. Moreover, multivariate analysis revealed that joint effusion could worsen the disc status. This probably resulted from inflammatory activity.

Although no uniform agreement exists regarding treatment protocol for ADD, nonsurgical treatment for at least 6 months is generally advocated^17^. Interestingly, our study found that follow-up period was unrelated to changes in disc length and position. Some patients even suffered from serious deterioration of disc status with a relatively short interval. Thus, some intriguing questions were raised: Does it always need a 6-month nonsurgical treatment before performing disc repositioning? Would early disc repositioning be benefit for disc status and condylar? Some surgeons questioned the importance of disc position and function, because degeneration in the TMJ often come to a standstill, yet relieving pain and restriction of mouth opening were priorities^6^,^28^. However, it seems premature to promote the idea that disc position is insignificant, since a long-term deleterious effect of not treating disc position may occur, such as gross degenerative changes of osteoarthrosis, arrested mandibular growth, or relapse of orthodontic and orthognathic treatment^10^,^18^,^28^,^37^. According to our experience, disc repositioning can be accomplished successfully by arthroscopy or open surgery when the configuration and length of the disc are normal or close to normal. Besides, obvious condylar remodeling can be achieved after disc repositioning in the teenage patients^38^. Nevertheless, it is difficult to reposition the displaced disc if it is serious deformed or perforated, and its ability to promote condylar remodeling would be hindered. From our results, discs in the ADDWR group wouldn’t change much in disc lengths or displacement distances after a relatively long follow-up, on the contrary, discs of ADDWoR patients could become severely deformed even with a short interval. Therefore, we recommend that nonsurgical treatment, like splint therapy, can be implemented first for symptomatic ADDWR patients. While a reliable disc repositioning surgery should be taken into account in the ADDWoR patients as early as possible, especially for teenagers and those with U-shaped or V-shaped folded disc and joint effusion, before the disc severely deformed and shortened. On the other hand, if the disc has already severely shortened or shrunk into a rounded shape, nonsurgical treatment should be performed before considering condylar reconstruction with a costochondral graft or joint prosthesis.

There were some limitations in this study. First, a retrospective study is not as convincing as a prospective study. Next, the follow-up period was relatively short when compared with other studies on the natural course of disc displacement. Although our results consisted with those long-term investigations, we do not know when disc distortion would cease. Moreover, a larger sample size should be warranted to obtain more detailed and convincing age-stratified and follow-up-stratified outcomes. At last, occlusal changes were not taken into consideration.

## Conclusions

Changes in disc morphology in the ADDWR group were mainly enlargement of posterior band, thickness or mild folding, while greater deformation of the disk such as serious folded and shortened configuration was more prevalent in the ADDWoR group after follow-up. Secondly, discs were prone to become shorter and more anteriorly displaced in teenagers, type I and III morphologies, advanced Wilkes stages, or those with joint effusion. Last but not least, follow-up period seemed to be not critical for disc status changes, which reminds TMJ surgeons to perform disc repositioning surgery as soon for ADDWoR patients with risk factors, instead of implement nonsurgical surgery for 6 months that may lead to serious deterioration in disc status.

## Additional Information

**How to cite this article**: Hu, Y.K. *et al*. Changes in disc status in the reducing and nonreducing anterior disc displacement of temporomandibular joint: a longitudinal retrospective study. *Sci. Rep*. **6**, 34253; doi: 10.1038/srep34253 (2016).

## Figures and Tables

**Table 1:** Inclusion and exclusion criteria for selection of patients.

Inclusion criteria	Exclusion criteria
Patients that	Patients
1) visited our clinic between January 2013 to December 2015 without sex or age restrictions;	1) with ADDWR becaming ADDWoR after follow-up;
2) had ADDWR or ADDWoR confirmed by MRI at first visit;	2) with poor image quality of MRI which was unsuitable for quantitative measurement, owing to movement by the patient when undergoing MRI.
3) had 2 MRI records with an interval longer than 3 months	
4) had no treatment before and during the follow-up period except for drugs;	
5) had no history of infection, injuries to the jaws, or congenital and systematic disorders.	

**Table 2:** Details of the study population.

	ADDWR		ADDWoR	
	N (joints)	%	N (joints)	%
Gender				
Male	21	24.4%	23	17.6%
Female	65	75.6%	108	82.4%
Age group (years)				
<15	21	24.4%	44	33.6%
15 ≤ age < 22	30	34.9%	53	40.5%
≥22	35	40.7%	34	25.9%
Follow-up period (months)				
3 ≤ F ≤ 6	32	37.2%	41	31.3%
6 < F ≤ 12	35	40.7%	85	64.9%
>12	19	22.1%	5	3.8%

**Table 3:**
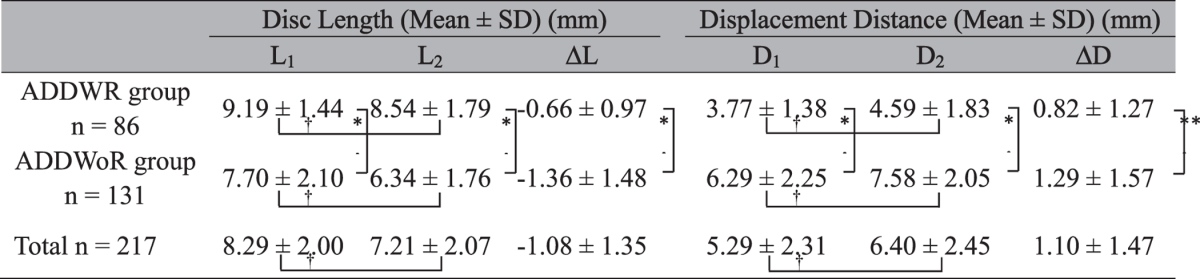
Changes in disc length and displacement distance.

^†^P < 0.001 (paired t-test).

*P < 0.001 (independent t-test).

**P = 0.017 (independent t-test).

**Table 4:** Changes in disc morphology and stages of ID.

	ADDWR			ADDWoR		
	Initial Visit N(%)	Follow-up visit N(%)	*P*	Initial Visit N(%)	Follow-up visit N(%)	*P*
Disc morphology						
Type I	59(68.6%)	45(52.3%)	0.007*	16(12.2%)	4(3.1%)	<0.001*
Type II	12(14.0%)	22(25.6%)		65(49.6%)	51(38.9%)	
Type III	15(17.4%)	19(22.1%)		21(16.0%)	16(12.2%)	
Type IV	0	0		25(19.1%)	53(40.5%)	
Type V	0	0		4(3.1%)	7(5.3%)	
Wilkes stages						
Stage I	69(80.2%)	43(50.0%)	<0.001*	0	0	<0.001*
Stage II	17(19.8%)	43(50.0%)		0	0	
Stage III	0	0		99(75.6%)	51(38.9%)	
Stage IV	0	0		30(22.9%)	74(56.5%)	
Stage V	0	0		2(1.5%)	6(4.6%)	

^*^Significant difference between initial and follow-up visits (Wilcoxon signed rank test).

**Table 5:** Effusion changes at initial and follow-up visits.

	Initial Visit			Follow-up Vist		
	ADDWR	ADDWoR	*P*	ADDWR	ADDWoR	*P*
Effusion						
^ ^Absent	78(90.7%)	109(83.2%)	0.118	86(100%)	127(96.9%)	0.263
^ ^Present	8(9.3%)	22(16.8%)		0(0)	4(3.1)	

**Table 6:** Univariate analysis for the influence of candidate contributing factors.

	∆L		∆D	
	Mean ± SD (mm)	*P*	Mean ± SD (mm)	*P*
Gender				
Male	−1.60 ± 1.61	0.005*	1.80 ± 1.47	<0.001*
Female	−0.95 ± 1.24		0.93 ± 1.42	
Age (y)				
<15	−1.64 ± 1.30	<0.001*	1.81 ± 1.47	<0.001*
15 ≤ age<22	−1.02 ± 1.17		0.90 ± 1.19	
≥ 22	−0.63 ± 1.42		0.67 ± 1.55	
Follow-up period (m)				
3 ≤ F ≤ 6	−1.19 ± 1.38	0.645	1.14 ± 1.19	0.272
6 < F ≤ 12	−1.01 ± 1.27		1.09 ± 1.62	
>12	−1.11 ± 1.61		1.13 ± 1.42	
Disc morphology				
Type I	−0.98 ± 1.37	0.006*	.97 ± 1.36	<0.001*
Type II	−1.36 ± 1.52		1.48 ± 1.63	
Type III	−1.27 ± 1.10		1.39 ± 1.40	
Type IV	−0.47 ± 0.73		0.16 ± 0.71	
Type V	0.03 ± 0.49		−0.40 ± 0.94	
Wilkes stages				
Stage I	−0.62 ± 0.86	<0.001*	0.83 ± 1.27	0.046*
Stage II	−0.79 ± 1.32		0.80 ± 1.31	
Stage III	−1.58 ± 1.47		1.42 ± 1.52	
Stage IV	−0.74 ± 1.33		1.01 ± 1.62	
Stage V	0.22 ± 0.77		−0.18 ± 0.16	
Joint effusion				
Absent	−0.93 ± 1.25	<0.001*	0.98 ± 1.43	0.001*
Present	−2.04 ± 1.52		1.90 ± 1.51	

^*^Significant difference by univariate analysis.

**Table 7:** Multivariate analysis for disc length changes.

	B	95% CI		P
		Lower	Upper	
Age (y)				
<15				
15 ≤ age < 22	0.674	0.271	1.077	0.001*
≥22	0.795	0.370	1.219	<0.001*
Disc morphology				
Type I				
Type II	0.551	0.071	1.031	0.025*
Type III	0.394	−0.123	0.912	0.135
Type IV	1.496	0.827	2.166	<0.001*
Type V	1.534	0.231	2.837	0.021*
Wilkes stages				
Stage I				
Stage II	−0.026	−0.677	0.626	0.938
Stage III	−1.132	−1.597	−0.667	<0.001*
Stage IV + V	−0.972	−1.616	−0.328	0.003*
Joint effusion				
Present				
Absent	0.786	0.294	1.279	0.002*

^*^Significant difference by multivariate analysis.

**Table 8:** Multivariate analysis for displacement distance changes.

	B	95% CI		P
		Lower	Upper	
Gender				
Female				
Male	0.519	0.047	0.992	0.031*
Age (y)				
<15				
15 ≤ age < 22	−0.990	−1.436	−0.545	<0.001*
≥22	−0.875	−1.355	−0.394	<0.001*
Disc morphology				
Type I				
Type II	−0.262	−0.788	0.263	0.327
Type III	−0.111	−0.678	0.457	0.701
Type IV	−1.634	−2.382	−0.887	<0.001*
Type V	−1.421	−2.849	0.006	0.051
Wilkes stages				
Stage I				
Stage II	−0.452	−1.188	0.284	0.227
Stage III	0.679	0.171	1.187	0.009*
Stage IV + V	0.993	0.283	1.702	0.006*
Joint effusion				
Present				
Absent	−0.603	−1.143	−0.062	0.029*
